# Indices of Mediterranean diet adherence and breast cancer risk in a community-based cohort

**DOI:** 10.3389/fnut.2023.1148075

**Published:** 2023-03-21

**Authors:** Ioanna Yiannakou, Martha R. Singer, Lynn L. Moore

**Affiliations:** ^1^Preventive Medicine and Epidemiology, Department of Medicine, Boston University Chobanian & Avedisian School of Medicine, Boston, MA, United States; ^2^Graduate Medical Sciences/Doctoral Program in Biomedical Sciences, Nutrition and Metabolism, Boston University Chobanian & Avedisian School of Medicine, Boston, MA, United States

**Keywords:** breast cancer, Mediterranean diet, dietary index, prospective cohort study, diet quality, hormone receptor status

## Abstract

**Introduction:**

A Mediterranean-style dietary pattern is believed to have cancer-protective effects. We compared the prospective associations between adherence to four established Mediterranean diet indices and breast cancer risk (including total, postmenopausal, and hormone receptor positive cases) in women in the Framingham Offspring Study.

**Methods:**

The four indices used two different approaches to measuring adherence to a Mediterranean diet: (a) scores based on the population-specific median intakes of Mediterranean diet-related foods in a given population (i.e., alternate Mediterranean Diet (aMED) index and Mediterranean Diet Score (MDS) index), and (b) scores based on compliance with recommended intakes of relevant foods from the Mediterranean diet pyramid [i.e., Mediterranean Diet (MeDiet) index and Mediterranean Style Dietary Pattern (MSDP) index]. Dietary data were derived from semiquantitative food frequency questionnaires collected in 1991-95. Participants included 1579 women aged ≤ 30 years who were free of prevalent cancer. Women were followed through 2014, and Cox proportional-hazards models were used to estimate hazard ratios (HRs) and 95% confidence intervals (CIs), adjusting for various confounders.

**Results:**

During a median follow-up of approximately 18 years, 87 breast cancer cases were documented. Women in the highest (vs. lowest) score category of the pyramid-based scores (i.e., MeDiet or MSDP) had approximately 45% statistically significantly lower breast cancer risks. These effects were even stronger for any hormone receptor positive cases using the MeDiet index (highest vs. lowest score categories: HR = 0.45, 95% CI: 0.22–0.90). Neither of the median intake-based scores (i.e., aMED, MDS) was associated with breast cancer risk.

**Discussion:**

Our results suggest that the methodology and the composition of Mediterranean diet indices influence their ability to assess conformity to this specific diet pattern and predict breast cancer risk.

## 1. Introduction

Dietary guidelines in the US recommend the Mediterranean diet as a healthy dietary pattern for the prevention of chronic disease risk, including cancer (1). The Mediterranean diet is a well-balanced plant-based diet. However, its specific definition varies somewhat due to the variations in culture, religion, ethnicity, and socio-economic status among and within the different Mediterranean countries (2). Nevertheless, a Mediterranean diet is commonly characterized by frequent consumption of non-refined grains, vegetables, fruits, nuts, olive oil, and dairy products, and moderate intakes of fish, poultry, potatoes, legumes, eggs, and sweets. Finally, moderate intake of red wine during meals and increased physical activity are important components of the Mediterranean lifestyle (3, 4).

Various *a priori* criterion-based dietary indices have been developed over the years to assess a population's conformity to the Mediterranean diet (5, 6) and examine the cancer-protective effects of this dietary pattern. Evidence from some observational studies suggests that higher adherence to the Mediterranean diet is protective against breast cancer (7), especially in postmenopausal women (8–10), although evidence on molecular subtypes (defined by hormone receptor status) is very limited and inconsistent (11–14).

Mediterranean diet indices are designed to assess conformity to a single dietary pattern, but they often have different dietary components, use different methodologies for assigning points and scoring the diet, and apply different weighting strategies (5, 6, 15). These differences may influence the ability of a given index to assess conformity to the diet and predict disease risk.

Over the last decades, more than 25 Mediterranean diet indices with different scoring methods were developed. Historically, the first Mediterranean diet index was created by Trichopoulou and her colleagues in 1995 (16). Then Trichopoulou and colleagues created a modified index in 2003 called the Mediterranean Diet Score (MDS) that excluded potatoes, added fish, and changed the alcohol score (17). Shortly thereafter, in 2005, an alternative index was created called the alternate Mediterranean Diet (aMED) index (18). The differences between the MDS and aMED were mainly in the selection of food groups (e.g., inclusion or exclusion of dairy, whole grains, fruit, and nuts). In our comparison of Mediterranean diet indices for this study, we propose first to examine these two indices of Mediterranean diet adherence that have similar scoring systems based on population-specific median intakes of Mediterranean diet-related foods in a given population. Since these scores may perform differently in non-Mediterranean populations who consume very different amounts of Mediterranean-style foods, we chose to compare these indices with two additional indices that base their scoring on adherence to recommended intakes of foods from the traditional Mediterranean diet pyramid (19). Thus, for these analyses, we chose to additionally evaluate the Mediterranean Diet (MeDiet) index developed by Panagiotakos and his colleagues in 2006 (20), as well as another index developed for use in the Framingham Study in 2009, called the Mediterranean Style Dietary Pattern (MSDP) index (21). These latter indixes may address some of the limitations of previous indices when used in non-Mediterranean populations. Whether these pyramid-based indices better predict disease risk than those based on population-specific median intakes has not been previously examined within the same study population.

This study aims to compare the prospective associations between adherence to four established Mediterranean diet indices and breast cancer risk in adult females in the Framingham Offspring Study (FOS). The four indices represent two different approaches to measuring adherence to the diet pattern: (a) scores based on the population-specific median intakes (i.e., MDS and aMED); and (b) scores based on compliance with recommended intakes (i.e., MeDiet and MSDP). In addition, since diet may act differently in preventing certain cancer subtypes (e.g., based on hormone receptor status) (22), we also propose to assess the associations between Mediterranean diet adherence and breast cancer risk stratified by hormone receptor status.

## 2. Material and methods

### 2.1. Study sample

In 1971, the Framingham Offspring Study enrolled the offspring and spouses of the original cohort members who had been followed for approximately four decades (23). Participants were asked to complete questionnaires on medical, demographic, diet, and other lifestyle information approximately every 4 years. The first complete food frequency questionnaire (FFQ) was administered at examination visit 5 (1991–1995), and that visit will serve as the baseline for these analyses; follow-up will continue until 2013, the last year with available cancer follow-up data. From a total of 3,712 subjects attending the fifth examination, we excluded the following subjects: (a) missing or invalid FFQ data (e.g., reported energy intakes < 600 kcal/d or >4,000 kcal/d) or 12 blank food items or missing data on calorie contribution from each food item (*n* = 362); (b) history of prevalent cancer, except non-melanoma skin cancer cases (*n* = 147); (c) age < 30 years (*n* = 4); (d) males (*n* = 1,496); and (e) missing covariates (*n* = 124). For analyses of estrogen or progesterone receptor (ER/PR) positive breast cancer cases, we additionally excluded 12 cases missing information on hormone receptor status (study sample used = 1,567). The Institutional Review Board of Boston University School of Medicine approved the Framingham Offspring Study data collection and these analyses (Protocols H-32086 and H-32132).

### 2.2. Assessment of adherence to the Mediterranean diet

Dietary data were collected *via* the Harvard 126-food item semiquantitative FFQ (24). The FFQ consists of a list of foods with standardized serving sizes and assesses the frequency of consumption during the previous year, ranging from “never or < one serving/month” to” ≥six servings/day.” Separate questions allowed subjects to add up to 3 additional foods usually consumed regularly that were not listed on the FFQ, as well as types of cold breakfast cereals and cooking oils usually used.

The MDS and aMED indices were chosen for these analyses as two of the most frequently used instruments that rely on median food intakes in the population. The MeDiet and MSDP indices were chosen as two indices reflecting adherence to recommended intakes of relevant foods from the Mediterranean diet pyramid. The food items included in each score component are shown in Supplementary Table S1, while the scoring standards and points to be assigned for these four indices are shown in Table 1. The serving sizes used for dietary variables in the two earlier indices (aMED and MDS) are taken from the FFQ. Serving sizes for the components in the MeDiet index were adjusted to match the recommended servings of the food guide pyramid (19).

**Table 1:** Standards for maximum scores on each Mediterranean diet index.

	**Standards for maximum scores**			
	**Median intake-based scores**		**Pyramid-based scores**	
**Individual components**	**MDS index** ^a^	**aMED index** ^a^	**MeDiet index** ^b^	**MSDP index** ^c^
Vegetables except potatoes	≥median (svgs/day)^d^	≥median (svgs/day)^d^	≥33 svgs/week^e^	6 svgs /day
Potatoes	—	—	≥4 svgs/week^e^	3 svgs/week
Legumes	≥median (svgs/day)^d^	≥median (svgs/day)^d^	≥6 svgs/week^e^	—
Olives, pulses, and nuts	—	—	—	4 svgs/week
Fruits, fruit juice and nuts	≥median (svgs/day)^d^	—	—	—
Fruits and fruit juice	—	≥median (svgs/day)^d^	≥22 svgs/week^e^	3 svgs/day
Nuts	—	≥median (svgs/day)^d^	—	—
Cereals	≥median (svgs/day)^d^	—	—	—
Wholegrains	—	≥median (svgs/day)^d^	—	8 svgs/day
Wholegrains, nuts, and seeds	—	—	≥32 svgs/week^e^	—
Dairy (low and full fat)	< median (svgs/day)^d^	—	—	2 svgs/day
Full fat dairy products	—	—	≤ 10 svgs/week	—
Fish	≥median (svgs/day)^d^	≥median (svgs/day)^d^	≥6 svgs/week^e^	6 svgs/week
Meat and meat products	< median (svgs/day)^d^	—	—	—
Red and processed meats	—	< median (svgs/day)^d^	—	1 svg/week
Red, processed meats and eggs	—	—	≤ 1 svgs/week	—
Poultry	—	—	≤ 3 svgs/week^e^	4 svgs/week
Eggs	—	—	—	3 svgs/week
Alcohol (grams/day)	5–25 grams/day (females)	5–15 grams/day	—	—
Alcoholic beverages	—	—	1– < 3 svgs/day^f^	—
Wine	—	—	—	1.5 svgs/day (females)
Mono:saturated fat ratio	≥median ratio	—	—	—
Mono:saturated fat ratio^g^	—	≥median ratio	—	—
(energy-adj)				
Olive oil	—	—	Olive oil only^h^	Olive oil only^i^
Sweets	—	—	—	3 svgs/week
**Total score**	0–9	0–9	0–55	0–100

MDS, Mediterranean Diet Score index by Trichopoulou et al., (17); aMED, Alternate Mediterranean Diet index by Fung et al., (18); MeDiet, Mediterranean Diet index by Panagiotakos et al., (20); MSDP, Mediterranean-Style Dietary Pattern index by Rumawas et al., (21); svgs, servings; mono, monounsaturated, and adj, adjusted.

^a^1 point for meeting criteria, 0 points if not.

^b^Standards reflect recommended intakes. Each component has 6 recommended intake categories (22), except the olive oil component, which has 3. The scoring for each component ranges from 0 to 5.

^c^Standards reflect recommended intakes. Each of the 13 food items except olive oil was scored from 0 to 10 depending on the degree of correspondence with the % of the recommended intake of that item. Then a penalty for overconsumption and a weighting factor for consumption of non-Mediterranean foods were applied.

^d^Servings were as listed on the food-frequency questionnaire.

^e^Servings were adjusted based on the food guide pyramid recommendations (5).

^f^1 serving of alcoholic beverage consists of 12 % ethanol.

^g^Dietary fats were adjusted for energy intake by adding residuals from linear regression models to overall median intake values.

^h^Olive oil consumption was classified into three categories: a) exclusive use of olive oil (score 5), use of olive oil along with other vegetable oils (score 3), or no olive oil (score 0).

^i^Exclusive use of olive oil score was given a score of 10 while the use of olive oil plus other vegetable oils received a score of 5, and no use of olive oil, a score of 0.

#### 2.2.1. Mediterranean Diet Score index (MDS)

The nine components of the MDS index (17), as shown in Table 1, are each assigned a value of 0 or 1. For the intake of vegetables (except potatoes), legumes, fruit, fruit juice and nuts, cereals, and fish, those participants whose intake was at or above the sex-specific median received a score of 1 (vs. a score of 0 for intakes below the median). Similarly, having a monounsaturated to saturated fat ratio ≥median (vs. < median) was also given a score of 1. For components believed to be less healthy (i.e., dairy and meat), subjects whose intake was below the median were assigned a value of 1 (vs. a score of 0 for ≥median). Subjects (females only in this study) with alcohol intake of 5–25 g/day were assigned a value of 1, while both lower and higher intakes received a score of 0. The total MDS ranged from 0 to 9.

#### 2.2.2. Alternate Mediterranean Diet index (aMED)

The aMED index (18) is a modified version of the MDS index (17). The aMED index separates fruits and nuts into two groups, eliminates the dairy group, includes whole grains instead of all cereals, and replaces all meats with red and processed meats. Further, alcohol intake for women was limited to 5–15 g/day to receive 1 point. Finally, the monounsaturated to saturated fat ratio was adjusted for energy using the residual method. The possible scores on the aMED index ranged from 0 to 9.

#### 2.2.3 Mediterranean Diet index (MeDiet)

The MeDiet score created by Panagiotakos et al. (20) ranges from 0 to 55 points, with up to 5 points being given for each of 11 items. Foods derived from mixed dishes and foods listed by participants were also assigned to the appropriate component scores. Scores of 0 to 5 were assigned to intakes of each healthy food component. For example, intakes of fruit (and fruit juice) in the following categories of weekly intake were given scores of 0–5, respectively: never, 1–4, 5–8, 9–15, 16–21, and ≥22 servings/week. Similar scoring was used for intakes of the following: 1) vegetables (except potatoes), 2) potatoes, 3) legumes, 4) wholegrain, nuts, and seeds, and 5) fish. Scores were reversed for full-fat dairy products, poultry, and red and processed meats (including eggs). For alcohol consumption, a score of 5 was assigned to drinkers with intakes of 1- < 3 servings (each serving is equal to 12 g of ethanol) per day and a score of 0 for < 1 or ≥3 servings per day. We modified the original scoring of olive oil due to the absence of information about the quantity of oil consumed on our FFQ. We classified olive oil consumption into three categories: a) exclusive use of olive oil (score 5), use of olive oil along with other vegetable oils (score 3), or no olive oil (score 0).

#### 2.2.4 Mediterranean-Style Dietary Pattern index (MSDP)

The fourth index used in this study is the MSDP (21) which is based on the recommended intake of 13 foods shown in a traditional Mediterranean diet pyramid (Table 1). All items on the FFQ, including mixed dishes, were evaluated to determine whether the item fits into one of the score components. Some foods (e.g., yams) were not traditionally consumed in Mediterranean regions. Still, if other similar foods (e.g., other potatoes) were consumed, the item (yams) was added to the score component (potatoes). Each of the 13 components except olive oil was scored from 0 to 10 depending on the degree of correspondence with the percent of the recommended intake of that item (e.g., consuming 40% of the recommended servings would result in a score of 4). Exceeding the recommendations resulted in a lower score proportional to the degree of overconsumption (e.g., exceeding the recommendation by 40% would result in a score of 6). A negative score due to more than 100% overconsumption was given a score of zero. Exclusive use of olive oil was given a score of 10, while the use of olive oil plus other vegetable oils received a score of 5, and no use of olive oil a score of 0. The sum of the 13 component scores was standardized on a scale of 0–100 and weighted (from 0 to 1) by the proportion of energy intake attributed to the consumption of foods included in the Mediterranean diet pyramid (e.g., if 45% of energy was derived from foods not included in the Mediterranean diet pyramid, the calculated weighting factor would be 0.55). The final total MSDP score has a theoretical range of 0–100.

### 2.3. Cancer outcomes

Framingham investigators used standardized protocols (25) to detect cancer cases using information from self-reports, surveillance of local hospital admissions, and searches of the state health department's death records and the National Death Index. The cancer diagnosis and ER/PR status for cases were validated based on information from pathology reports and, in a few instances, from medical records. Breast cancer cases were defined using the International Classification of Diseases (ICD-O-3). In total, there were 87 first primary-site malignant breast cancer cases.

### 2.4. Potential confounders

We assessed several potential confounders at baseline, including age, cigarette smoking, alcohol intake, supplement use, energy intake, parity, body mass index (BMI), waist-to-height ratio (WHtR), and type 2 diabetes mellitus (T2DM). We also used information from all the examination visits to determine age at menopause and estrogen use. Self-reported information about education level was derived from the first exam in FOS and used to classify participants as having a college or graduate degree vs. less. An index for moderate and vigorous physical activity (in metabolic equivalents per hour) was created previously based on self-reported data (26) at every exam except exam 6. Because the index distribution at baseline exam (exam 5) was highly skewed to the right compared to the rest exams, we used the mean of physical activity index from exams 4 and 7. Participants were diagnosed with T2DM at each exam based on standardized Framingham criteria as described previously (27). For baseline anthropometric measures, a standard beam balance (with a stadiometer) was used to measure weight and height with the subject dressed in a hospital gown with no shoes. Because of the natural height loss occurring after age 60, we calculated the average of all height measures for adults up to age 60. Waist circumference was measured at the level of the umbilicus during mid-respiration to the nearest 0.25 inch with a cloth tape. Waist circumference was divided by average height measure to calculate WHtR (with missing values at exam 5 substituted using the mean values from exams 4 and 6).

Postmenopausal women were asked to report their age at menopause directly. In addition, at each exam, women were asked about their current menopausal status and estrogen use. For those missing self-reported age at menopause, we took the age at the exam at which a woman first reported being postmenopausal to be her age at menopause. Otherwise, we took the age at which a woman first reported using estrogen (typically for perimenopausal symptoms) and added 1 year to estimate the age at menopause. For non-estrogen users aged < 50 years old, we substituted age at menopause with the median age of natural menopause in this cohort (50 years). Lastly, for non-estrogen users who were ≥50 years old, we took the age at menopause to be the age at the exam at which a woman last reported being premenopausal plus 1 year.

Estrogen use was treated as a time-dependent variable and classified as never or ever use (prior to, at baseline, or during the follow-up period). Women with missing information on estrogen use who reported no use at any other exams were treated as non-users for the missing exams.

### 2.5. Statistical analysis

Descriptive statistics were used to describe the total score distribution of each index. Pearson correlation coefficients were computed to compare total scores on the four Mediterranean diet indices. As previously done (17), we classified total scores of both MDS and aMED indices to reflect low, moderate, and high categories of adherence to the Mediterranean diet as follows: 0–3, 4–5, and 6–9 points, respectively. Sensitivity analyses and the score distributions were used to determine the cut-off values for classifying the final two indices into three categories: MeDiet index (low: 14–28; moderate: 29–34; and high: 35–53) and MSDP index (low: 4.0–19.0; moderate: 19.1–25.0; high: 25.1–50.9). The lowest category was used as the referent group for all analyses.

Breast cancer outcomes included total incident cases, postmenopausal cases, and any ER or PR positive cases (including ER-/PR+, ER+/PR-, ER+/PR+). Incidence rates for breast cancer were computed in each diet score category using person-years of follow-up calculated from exam 5 to the first of the following events: occurrence of incident breast cancer, loss of follow-up, date of the last exam, or date of death. Survival analyses were conducted to examine the association between adherence to each of the four Mediterranean diet indices and breast cancer risk. Cox proportional hazards regression models were used to estimate adjusted hazard ratios (HRs) and 95% CIs for the occurrence of first breast cancer cases. Tests for linear trends across categories of scores were performed using the category-specific medians in each score category.

The basic model for each index was adjusted for age (in years) and total calorie intake; except MSDP adjusted for age only because MSDP score accounts for calorie intake in its scoring system. Next, we examined a list of potential confounders and evaluated how they altered the basic model-parameter estimates by approximately 10% or more. We adjusted for WHtR, cigarette smoking (pack years), physical activity (METs/hour), diabetes status, supplement use, and age at menopause. Factors that were found not to confound the association between adherence to the Mediterranean diet and breast cancer risk were not included in the final model, i.e., alcohol intake and educational level. Lastly, highly collinear variables were not included together in the model, i.e., age at menopause and estrogen use. Finally, the proportional hazards assumptions were tested by using an interaction term with time in the models. No violations of the assumption were found. Statistical analyses were conducted using SAS statistical software (version 9.4; SAS Institute, Cary, NC).

## 3. Results

### 3.1. Descriptive information

All Mediterranean diet scores were normally distributed in the FOS cohort. The mean (standard deviation, SD) scores for the MDS and aMED were very similar, 4.2 (1.7) and 4.3 (1.9) out of the possible maximum score of 9, respectively. Mean scores (SD) for MeDiet and MSDP scores were 32.1 (5.2) out of a hypothetical maximum possible score of 55 and 23.4 (7.2) out of a hypothetical maximum score of 100, respectively.

Table 2 shows the baseline characteristics of the participants according to the score categories for each Mediterranean diet index. Overall, women in the highest score category for all indices were older, more frequently had a high school degree or above, were more active, and smoked fewer cigarettes compared with those in the lowest category. Further, women with higher adherence to the Mediterranean diet (higher score categories) consumed more calories and were more likely to take dietary supplements.

**Table 2:** Baseline characteristics^a^ across the score categories of Mediterranean diet indices in women of the Framingham Offspring Study.

**Mediterranean diet indices**	**Age (years)**	**BMI (kg/m^2^)**	**Waist (cm)**	**Cigarettes (pack years)**	**Calorie intake (kcals/day)**	**Supplement use, %**	**Physical activity (METs/hour)**	**Age at menopause^b^ (years)**	**Estrogen users^c^, %**	**Prevalent diabetes, %**	**≥High school, %**
**Median intake-based scores**											
**MDS index**											
Low (0–3)	52.2 (9.4)	26.9 (5.8)	87.6 (15.2)	15.4 (1.0)	1,614.8 (534.6)	27.0	13.5 (7.7)	47.9 (5.8)	45.1	4.3	20.4
Moderate (4–5)	54.7 (9.6)	26.7 (5.5)	87.1 (14.7)	12.2 (0.8)	1,766.2 (592.1)	33.1	14.3 (7.3)	48.0 (6.3)	47.0	5.4	29.6
High (6–9)	56.4 (9.0)	25.9 (4.8)	86.0 (13.4)	9.6 (0.9)	1,929.1 (532.7)	39.7	15.2 (7.3)	48.5 (5.3)	48.7	4.6	30.8
**aMED index**											
Low (0–3)	53.1 (9.5)	26.9 (5.9)	87.6 (15.5)	16.4 (1.1)	1,566.6 (549.9)	27.1	13.8 (7.9)	47.8 (5.9)	44.5	5.1	20.1
Moderate (4–5)	54.2 (9.7)	26.6 (5.2)	87.0 (13.9)	11.6 (0.8)	1,746.3 (538.9)	32.2	13.7 (7.0)	48.1 (6.2)	47.4	4.3	28.4
High (6–9)	55.5 (9.2)	26.2 (5.1)	86.2 (14.3)	9.6 (0.8)	1,993.3 (547.5)	39.8	15.4 (7.5)	48.5 (5.6)	48.7	5.1	32.6
**Pyramid-based scores**											
**MeDiet index**											
Low (15–28)	53.3 (9.8)	27.2 (5.6)	88.9 (15.6)	17.2 (1.3)	1,597.8 (601.5)	27.4	13.7 (8.2)	47.9 (5.7)	41.0	4.0	21.5
Moderate (29–34)	54.2 (9.5)	26.7 (5.5)	87.3 (14.4)	13.6 (0.8)	1,726.7 (545.3)	31.4	13.9 (7.1)	47.7 (6.3)	48.4	6.3	24.9
High (35–51)	54.8 (9.2)	25.9 (5.2)	85.2 (13.9)	8.8 (0.7)	1,901.6 (540.7)	37.9	15.0 (7.4)	48.8 (5.4)	48.9	3.4	32.9
**MSDP index**											
Low (4.0–19.0)	52.8 (9.4)	26.5 (5.7)	87.0 (14.9)	17.7 (1.2)	1,591.6 (595.3)	25.8	13.4 (7.9)	47.7 (5.9)	46.4	5.2	21.3
Moderate (19.1–25.0)	54.6 (9.9)	26.8 (5.3)	87.6 (14.6)	12.7 (0.9)	1,777.6 (569.9)	27.9	14.1 (7.2)	48.1 (5.7)	42.9	4.2	25.7
High (25.1–50.9)	54.8 (9.2)	26.5 (5.4)	86.6 (14.4)	9.6 (0.7)	1,836.1 (529.4)	40.7	14.8 (7.3)	48.3 (6.1)	49.8	5.1	30.9

BMI, body mass index; SE, standard error; METs, metabolic equivalents; MDS, Mediterranean Diet Score index by Trichopoulou et al., (17); aMED, Alternate Mediterranean Diet index by Fung et al., (18); MeDiet, Mediterranean Diet index by Panagiotakos et al., (20); MSDP, Mediterranean-Style Dietary Pattern index by Rumawas et al., (21).

^a^Values for continuous variables are arithmetic means (standard deviation) except for cigarette smoking which is a geometric mean.

^b^Including natural and non-natural menopause.

^c^Percent of women ever using estrogen from exams 1 to 8, of which 256 were missing information. Sample used is 1,323 women.

Correlations between the four Mediterranean diet scores are shown in Supplementary Table S2. The strongest correlation was between the two scores based on the population-specific median intakes, MDS and aMED (*r* = 0.83). There were moderate to high correlations between MeDiet and all other scores (*r* = 0.57–0.61). Lastly, the MSDP score was only weakly correlated with MDS (*r* = 0.39) and moderately correlated with aMED (*r* = 0.54).

### 3.2. Adherence to the Mediterranean diet and breast cancer

Table 3 shows the associations between two median intake-based indices and two pyramid-based Mediterranean diet scores with the risk of breast cancer. Those in the high pyramid-based score categories of MeDiet and MSDP indices had the lowest cancer incidence rate, 2.42 and 2.46 per 1,000 person-years of follow-up, respectively. After adjusting for multiple confounders, women in the highest (vs. lowest) category of these two pyramid-based scores had approximately 45% lower breast cancer risks, both with statistically significant trends. We found no association between median intake-based scores (i.e., MDS and aMED scores) and breast cancer risk.

**Table 3:** Hazard ratios for breast cancer associated with Mediterranean diet indices.

**Mediterranean diet indices**		***N***	**Cases**	**Rate per 1,000 PY**	**Age and calorie-adjusted HR (95% CI)^a^**	**Multivariable HR (95% CI)^b^**
**Median intake-based scores**						
**MDS index**						
	Low (0–3)	578	32	3.15	1 (Ref)	1 (Ref)
	Moderate (4–5)	631	31	2.80	0.83 (0.51–1.38)	0.85 (0.51–1.41)
	High (6–9)	370	24	3.75	1.08 (0.62–1.87)	1.15 (0.65–2.01)
	*P*−−*trend*^c^				*0.70*	*0.55*
**aMED index**						
	Low (0–3)	568	33	3.33	1 (Ref)	1 (Ref)
	Moderate (4–5)	581	30	2.94	0.91 (0.68–2.21)	0.89 (0.53–1.47)
	High (6–9)	430	24	3.19	0.93 (0.67–1.29)	0.91 (0.51–1.60)
	*P*−−*trend*^c^				*0.58*	*0.71*
**Pyramid-based scores**						
**MeDiet index**						
	Low (15–28)	401	30	4.31	1 (Ref)	1 (Ref)
	Moderate (29–34)	682	36	3.01	0.67 (0.41–1.09)	0.68 (0.42–1.12)
	High (35–51)	496	21	2.42	0.52 (0.29–0.91)	0.55 (0.31–0.97)
	*P*−−*trend*^c^				*0.02*	*0.04*
**MSDP index**						
	Low (4.0–19.0)	442	32	4.21	1 (Ref)	1 (Ref)
	Moderate (19.1–25.0)	506	27	3.05	0.69 (0.50–0.94)	0.71 (0.43–1.19)
	High (25.1–50.9)	631	28	2.50	0.71 (0.53–0.95)	0.56 (0.33–0.94)
	*P*−−*trend*^c^				*0.03*	*0.03*

PY, person-years; MDS, Mediterranean Diet Score index by Trichopoulou et al., (17); aMED, Alternate Mediterranean Diet index by Fung et al., (18); MeDiet, Mediterranean Diet index by Panagiotakos et al., (20); MSDP, Mediterranean-Style Dietary Pattern index by Rumawas et al., (21).

^a^Model adjusted for age and total calorie intake. MSDP score analysis was adjusted for age only.

^b^Model was additionally adjusted for waist-to-height ratio, cigarette smoking (pack-years), physical activity, diabetes status, supplement use, and age at menopause.

^c^Derived from the test for linear trend, modeling the median value for each category as a continuous variable. Two-sided *P*-values < 0.05 indicated statistical significance.

We further analyzed the associations between the four Mediterranean diet indices and breast cancer risk among postmenopausal women only (Table 4). Due to insufficient power, we could not analyze premenopausal breast cancer separately, as there were only 6 cases of premenopausal breast cancer. Both pyramid-based scores (MeDiet and MSDP indices) were inversely associated with postmenopausal breast cancer risk; however, the results did not reach statistical significance. Once again, neither the MDS nor the aMED scores were associated with postmenopausal breast cancer risk.

**Table 4:** Hazard ratios for postmenopausal breast cancer associated with Mediterranean diet indices.

**Mediterranean diet indices**		***N***	**Cases**	**Rate per 1,000 PY**	**Age and calorie-adjusted HR (95% CI)^a^**	**Multivariable HR (95% CI)^b^**
**Median intake-based scores**						
**MDS index**						
	Low (0–3)	578	29	2.85	1 (Ref)	1 (Ref)
	Moderate (4–5)	631	28	2.52	0.80 (0.47–1.36)	0.81 (0.48–1.38)
	High (6–9)	370	24	3.75	1.13 (0.64–1.98)	1.18 (0.66–2.09)
	*P*−−*trend*^c^				*0.55*	*0.46*
**aMED index**						
	Low (0–3)	568	29	2.92	1 (Ref)	1 (Ref)
	Moderate (4–5)	581	28	2.74	1.04 (0.59–1.84)	0.92 (0.54–1.57)
	High (6–9)	430	24	3.19	0.93 (0.53–1.61)	0.99 (0.55–1.77)
	*P*−−*trend*^c^				*0.86*	*0.96*
**Pyramid-based scores**						
**MeDiet index**						
	Low (15–28)	401	27	3.86	1 (Ref)	1 (Ref)
	Moderate (29–34)	682	33	2.75	0.67 (0.40–1.12)	0.68 (0.41–1.15)
	High (35–51)	496	21	2.42	0.56 (0.32–1.01)	0.58 (0.32–1.06)
	*P*−−*trend*^c^				*0.05*	*0.07*
**MSDP index**						
	Low (4.0–19.0)	442	28	3.67	1 (Ref)	1 (Ref)
	Moderate (19.1–25.0)	506	26	2.94	0.77 (0.45–1.31)	0.76 (0.45–1.31)
	High (25.1–50.9)	631	27	2.41	0.62 (0.36–1.05)	0.59 (0.34–1.02)
	*P*−−*trend*^c^				*0.08*	*0.06*

PY, person-years, MDS, Mediterranean Diet Score index by Trichopoulou et al., (17); aMED, Alternate Mediterranean Diet index by Fung et al., (18); MeDiet, Mediterranean Diet index by Panagiotakos et al., (20); MSDP, Mediterranean-Style Dietary Pattern index by Rumawas et al., (21).

^a^Model adjusted for age and total calorie intake. MSDP score analysis was adjusted for age only.

^b^Model was additionally adjusted for waist-to-height ratio, cigarette smoking (pack-years), physical activity, diabetes status, supplement use, and age at menopause.

^c^Derived from the test for linear trend, modeling the median value for each category as a continuous variable. Two-sided *P*-values < 0.05 indicated statistical significance.

Lastly, we examined the prospective associations between the four Mediterranean diet indices and the risk of any ER/PR positive breast cancer (Table 5). The highest MeDiet score category was strongly and inversely associated with any positive ER/PR breast cancer risk compared with the lowest category (HR: 0.45, 95% CI: 0.22–0.90). Results for the MSDP were weaker. We observed no association between the median intake-based scores and any ER/PR positive breast cancer risk.

**Table 5:** Hazard ratios for any ER/PR positive breast cancer associated with Mediterranean diet indices.

**Mediterranean diet indices**		***N***	**Cases**	**Rate per 1,000 PY**	**Age and calorie-adjusted HR (95% CI)^a^**	**Multivariable HR (95% CI)^b^**
**Median intake-based scores**						
**MDS index**						
	Low (0–3)	575	25	2.46	1 (Ref)	1 (Ref)
	Moderate (4–5)	624	21	1.90	0.73 (0.40–1.31)	0.75 (0.41–1.35)
	High (6–9)	368	18	2.81	1.04 (0.55–1.96)	1.13 (0.59–2.16)
	*P*−−*trend*^c^				*0.78*	*0.60*
**aMED index**						
	Low (0–3)	565	26	2.62	1 (Ref)	1 (Ref)
	Moderate (4–5)	575	22	2.16	1.37 (0.71–2.63)	0.83 (0.46–1.49)
	High (6–9)	427	16	2.12	1.08 (0.56–2.08)	0.77 (0.39–1.50)
	*P*−−*trend*^c^				*0.33*	*0.42*
**Pyramid-based scores**						
**MeDiet index**						
	Low (15–28)	398	23	3.29	1 (Ref)	1 (Ref)
	Moderate (29–34)	675	28	2.35	0.68 (0.39–1.19)	0.70 (0.40–1.22)
	High (35–51)	494	13	1.49	0.42 (0.21–0.83)	0.45 (0.22–0.90)
	*P*−−*trend*^c^				*0.01*	*0.02*
**MSDP index**						
	Low (4.0–19.0)	438	24	3.15	1 (Ref)	1 (Ref)
	Moderate (19.1–25.0)	502	19	2.15	0.67 (0.36–1.22)	0.67 (0.37–1.23)
	High (25.1–50.9)	627	21	1.88	0.57 (0.32–1.03)	0.57 (0.31–1.04)
	*P*−−*trend*^c^				*0.07*	*0.07*

ER/PR, estrogen receptor or progesterone receptor; PY, person-years; MDS, Mediterranean Diet Score by Trichopoulou et al., (17); aMED, Alternate Mediterranean Score by Fung et al., (18); MeDiet, Mediterranean Diet Score by Panagiotakos et al., (20); and MSDP, Mediterranean–Style Dietary Pattern Score by Rumawas et al., (21).

^a^Model adjusted for age and total calorie intake. MSDP score analysis was adjusted for age only.

^b^Model was additionally adjusted for waist-to-height ratio, cigarette smoking (pack-years), physical activity, diabetes status, supplement use, and age at menopause.

^c^Derived from the test for linear trend, modeling the median value for each category as a continuous variable. Two-sided *P*-values < 0.05 indicated statistical significance.

## 4. Discussion

In this prospective cohort study of middle-aged women in a non-Mediterranean population, we examined the association between four different Mediterranean diet indices and breast cancer risk over approximately 18 years of follow-up. These four indices represent two different approaches to measuring adherence to a Mediterranean diet: (a) scores based on the population-specific median intakes of Mediterranean diet-related foods (i.e., aMED and MDS indices), and (b) scores based on compliance with recommended intakes of relevant foods from the Mediterranean diet pyramid (i.e., MeDiet and MSDP indices). We found that higher adherence to MeDiet and MSDP indices was protectively associated with incident breast cancer. The risk estimates from the pyramid-based scores were associated with more than a 40% reduction in risk of total breast cancer as well as postmenopausal breast cancer. Results for hormone-receptor positive breast cancer were similar for MeDiet. Neither of the scores based on the population-specific median intakes (i.e., MDS and aMED indices) was associated with incident breast cancer risk.

Previous studies conducted in non-Mediterranean populations have frequently used Mediterranean diet scores based on median intakes when examining breast cancer risk. Studies using the MDS or aMED indices showed no association between adherence to the Mediterranean diet and breast cancer risk (10, 11, 14, 28–32), results that are consistent with the present study. Although the original authors who developed the aMED index in the Nurses' Health Study found that higher scores were protective against several inflammatory biomarkers (18) and cardiometabolic diseases (33)—mechanisms that may be linked with the development of cancer—the scores were not predictive of postmenopausal breast cancer risk (14). However, they did find that aMED scores were inversely associated with ER- breast cancer risk among postmenopausal women in a non-linear fashion. Contrary to our results, analyses in two cohort studies in US women showed that higher aMED scores (without alcohol in the index) were associated with somehow lower total risks of ER+ (12) and postmenopausal breast cancer (9, 10) but these results did not reach statistical significance. The European Prospective Investigation into Cancer and Nutrition (EPIC) cohort study, which recruited 335,062 women from a mix of the Mediterranean and non-Mediterranean countries, showed that higher scores on a modified version of aMED index (excluding alcohol and some food groups) were associated with lower risk of postmenopausal but not premenopausal breast cancer (13).

We are the first to report on associations between pyramid-based scores (i.e., MSDP and MeDiet indices) and breast cancer risk in a non-Mediterranean population. We found that both of these two indices were inversely associated with breast cancer risk in this mainly American Caucasian population. The evidence using these scores to predict breast cancer risk in Mediterranean populations has been limited, and results were inconsistent, possibly due to the limitations associated with a case-control design (34, 35). Nevertheless, previous findings showed that higher adherence to these pyramid-based scores might prevent the development of metabolic dysfunction and, as a result, prevent the development of obesity-related cancers, including breast cancer. Previous prospective cohort analysis in the Framingham Study showed that individuals without prevalent diabetes who had higher MSDP scores had less metabolic dysfunction, as evidenced by a lower waist circumference, lower fasting plasma glucose, lower triglyceride levels, less insulin resistance, and higher HDL cholesterol levels, compared with those who had lower scores (36). Further, an analysis among US adults from the NHANES study showed that higher MeDiet scores were associated with lower adiposity measures, inflammatory markers, and glucose and lipoprotein a levels after multivariable adjustment (37). Moreover, the present study found that higher MeDiet scores were strongly and inversely associated with any hormone receptor (ER/PR) positive breast cancer risk. This could be due to effects of certain bioactive compounds (e.g., anti-oxidants and flavonoids) in the Mediterranean diet that have been shown to reduce endogenous estrogen production and increase sex-hormone binding globulin levels, thus decreasing circulating levels of estrogen (13).

Methodological differences in the composition of different indices may explain the inconsistency between studies with respect to the cancer-protective effects of the Mediterranean diet (15). Specifically, it may be that scores based on median cut-off values may perform differently in Mediterranean and non-Mediterranean populations as they consume different amounts of traditional Mediterranean-style foods. In contrast, scores that are based on recommended intakes of relevant foods may be more appropriately applied in different non-Mediterranean populations because the standards for assigning points do not change based on the intake distributions. Further, the foods considered to be Mediterranean foods differ between indices (e.g., potatoes are excluded from the MDS and aMED indices and dairy from the aMED). There are other differences between these indices as well. The MSDP score has a penalty for overconsumption and takes into account the consumption of non-Mediterranean foods of a similar type. However, these features make the score more computationally complex. Comparing the MeDiet and MSDP indices, the overconsumption penalty and the weighting strategies used to account for total energy intake in the MSDP index may explain the generally lower adherence (the maximum observed score in this study was 50.9 out of a possible maximum of 100 points) to the Mediterranean diet pattern with this index. In contrast, the MeDiet index applied in the same study population generally produced much higher adherence scores (a maximum of 51 out of a possible maximum of 55 points). Moreover, the standard for assigning the maximum points for each MSDP component score was based on a single recommended intake value, making it difficult for most individuals to be fully adherent to each component.

A systematic review comparing 28 Mediterranean diet indices concluded that the MeDiet index was one of the very few scores that provided strong construct and content validity, internal consistency, and evidence for an association with biological markers. The MSDP and MDS indices did not perform as well on these psychometric properties, and the aMED index was not evaluated (36). A more recent review comparing 8 different Mediterranean diet indices concluded that those indices whose scoring scheme was based on a single threshold (e.g., MDS), thus yielding a dichotomous score for each food item, provided less accurate measures of adherence to the diet pattern compared with others that assign scores proportional to the degree of concordance. While each index has strengths and limitations, the current findings suggest that the scores based on population-specific median intakes may not be as appropriate as the pyramid-based scores for measuring adherence to the Mediterranean diet in a non-Mediterranean population.

Some of the strengths of this study include the direct comparison of four established Mediterranean diet indices within the same non-Mediterranean study population to predict the same outcome. We used a prospective design and a thorough adjudication of cancer cases using standardized procedures. In addition, confounding effects were minimized as much as possible, given the careful, systematic collection of various variables. For example, adiposity, an important risk factor for breast cancer, was directly measured rather than self-reported. Nonetheless, residual confounding is always a possibility.

There are several limitations in this study as well. The dietary information was derived from FFQs, which have been associated with measurement errors. However, earlier validation studies of the Harvard FFQ showed that many of the foods included in these indices were adequately captured on the FFQ based on correlations with diet records (38). Another important limitation of this study is the limited power associated with small numbers of subjects, especially for the analysis of breast cancer subtypes. Further, although the use of oral contraceptives is a traditional breast cancer risk factor; these data were not available in this dataset. Lastly, the FOS cohort consists of exclusively Caucasian individuals, and therefore more studies are needed to confirm these results in other non-Mediterranean populations.

## 5. Conclusion

In this prospective study of middle-aged American Caucasian women, we found that higher adherence to the Mediterranean diet, as assessed by scores based on compliance with recommended intakes of relevant foods (MeDiet and MSDP indices) from the Mediterranean diet pyramid, was associated with a lower risk of incident breast cancer. This was not the case for scores based on population-specific median intakes of Mediterranean diet-related foods (MDS and aMED indices). In conclusion, our results suggest that the methodology and the composition of Mediterranean diet indices influence their ability to assess conformity to this specific diet pattern and predict disease risk. Specifically, indices based on median intakes may not predict cancer risk as well as scores based on traditional Mediterranean diet pyramid recommendations when used in non-Mediterranean populations. The heterogeneity in these indices and their use in different study populations may explain some of the differences in results between existing studies of the Mediterranean diet pattern and risk of breast cancer and other chronic diseases.

## Data availability statement

Publicly available datasets were analyzed in this study. This data can be found upon request at https://biolincc.nhlbi.nih.gov/studies/framoffspring/ (accessed on 13 September 2022).

## Ethics statement

The studies involving human participants were reviewed and approved by the Institutional Review Board of Boston University School of Medicine which approved the Framingham Offspring Study data collection and these analyses (Protocols H-32086 and H-32132). The participants provided their written informed consent to participate in this study.

## Author contributions

LLM and IY designed the analysis and wrote the manuscript. IY analyzed the data. MRS curated the data. IY, MRS, and LLM participated in interpreting the results and editing the manuscript. All authors read and approved the final manuscript.

## References

[1] U.S. Department of Agriculture and U.S. Department of Health and Human Services. Dietary Guidelines for Americans, 2020-2025. 9th ed. (2020). Available online at: DietaryGuidelines.gov (accessed February 20, 2023).

[2] Mattavelli E Olmastroni E Bonofiglio D Catapano AL Baragetti A Magni P. Adherence to the Mediterranean diet: impact of geographical location of the observations. Nutrients. (2022) 14:2040. 10.3390/nu14102040 35631181 PMC9144454

[3] Finicelli M Di Salle A Galderisi U Peluso G. The Mediterranean diet: an update of the clinical trials. Nutrients. (2022) 14:2956. 10.3390/nu14142956 35889911 PMC9317652

[4] Martínez-González MÁ Hershey MS Zazpe I Trichopoulou A. Transferability of the Mediterranean diet to non-Mediterranean countries. What is and what is not the Mediterranean diet. Nutrients. (2017) 9:1226. 10.3390/nu9111226 29117146 PMC5707698

[5] Chiriacò M Tubili C Bo S Parillo M Vetrani C Mazzotti A. Critical evaluation of the questionnaires assessing adherence to the Mediterranean diet that are based on servings. Nutr Metabol Cardiovasc Dis. (2023). 10.1016/j.numecd.2023.01.024. [Epub ahead of print]. 36842958

[6] Bach A Serra-Majem L Carrasco JL Roman B Ngo J Bertomeu I. The use of indexes evaluating the adherence to the Mediterranean diet in epidemiological studies: a review. Public Health Nutr. (2006) 9:132–46. 10.1079/PHN2005936 16512961

[7] Morze J Danielewicz A Przybyłowicz K Zeng H Hoffmann G Schwingshackl L. An updated systematic review and meta-analysis on adherence to Mediterranean diet and risk of cancer. Eur J Nutr. (2021) 60:1561–86. 10.1007/s00394-020-02346-6 32770356 PMC7987633

[8] Turati F Carioli G Bravi F Ferraroni M Serraino D Montella M. Mediterranean diet and breast cancer risk. Nutrients. (2018) 10:326. 10.3390/nu10030326 29518016 PMC5872744

[9] Haridass V Ziogas A Neuhausen SL Anton-Culver H Odegaard AO. Diet quality scores inversely associated with postmenopausal breast cancer risk are not associated with premenopausal breast cancer risk in the California Teachers Study. J Nutr. (2018) 148:1830–7. 10.1093/jn/nxy187 30247577

[10] Brandt PA. van den Schulpen M. Mediterranean diet adherence and risk of postmenopausal breast cancer: results of a cohort study and meta-analysis. Int J Cancer. (2017) 140:2220–31. 10.1002/ijc.30654 28260236

[11] Hirko KA Willett WC Hankinson SE Rosner BA Beck AH Tamimi RM. Healthy dietary patterns and risk of breast cancer by molecular subtype. Breast Cancer Res Treat. (2016) 155:579–88. 10.1007/s10549-016-3706-2 26872903 PMC4770904

[12] Petimar J Park Y-MM Smith-Warner SA Fung TT Sandler DP. Dietary index scores and invasive breast cancer risk among women with a family history of breast cancer. Am J Clin Nutr. (2019) 109:1393–401. 10.1093/ajcn/nqy392 30968114 PMC6499503

[13] Buckland G Travier N Cottet V González CA Luján-Barroso L Agudo A. Adherence to the Mediterranean diet and risk of breast cancer in the European prospective investigation into cancer and nutrition cohort study. Int J Cancer. (2013) 132:2918–27. 10.1002/ijc.27958 23180513

[14] Fung TT Hu FB McCullough ML Newby PK Willett WC Holmes MD. Diet quality is associated with the risk of estrogen receptor–negative breast cancer in postmenopausal women. J Nutr. (2006) 136:466–72. 10.1093/jn/136.2.466 16424129

[15] Hutchins-Wiese HL Bales CW Starr KNP. Mediterranean diet scoring systems: understanding the evolution and applications for Mediterranean and non-Mediterranean countries. Br J Nutr. (2021) 128:1–22. 10.1017/S0007114521002476 34289917

[16] Trichopoulou A Kouris-Blazos A Wahlqvist ML Gnardellis C Lagiou P Polychronopoulos E. Diet and overall survival in elderly people. BMJ. (1995) 311:1457–60. 10.1136/bmj.311.7018.1457 8520331 PMC2543726

[17] Trichopoulou A Costacou T Bamia C Trichopoulos D. Adherence to a Mediterranean diet and survival in a Greek population. N Engl J Med. (2003) 348:2599–608. 10.1056/NEJMoa025039 12826634

[18] Fung TT McCullough ML Newby PK Manson JE Meigs JB Rifai N. Diet-quality scores and plasma concentrations of markers of inflammation and endothelial dysfunction. Am J Clin Nutr. (2005) 82:163–73. 10.1093/ajcn.82.1.163 16002815

[19] Ministry of Health and Welfare Supreme Scientific Health Council of Greece. Dietary guidelines for adults in Greece. Arch Hellenic Med. (1999) 16:516–24.

[20] Panagiotakos DB Pitsavos C Stefanadis C. Dietary patterns: a Mediterranean diet score and its relation to clinical and biological markers of cardiovascular disease risk. Nutr Metab Cardiovasc Dis. (2006) 16:559–68. 10.1016/j.numecd.2005.08.006 17126772

[21] Rumawas ME Dwyer JT Mckeown NM Meigs JB Rogers G Jacques PF. The development of the Mediterranean-style dietary pattern score and its application to the American diet in the Framingham Offspring Cohort. J Nutr. (2009) 139:1150–6. 10.3945/jn.108.103424 19357215 PMC2682986

[22] Prat A Pineda E Adamo B Galván P Fernández A Gaba L. Clinical implications of the intrinsic molecular subtypes of breast cancer. The Breast. (2015) 24:S26–35. 10.1016/j.breast.2015.07.008 26253814

[23] Feinleib M Kannel WB Garrison RJ McNamara PM Castelli WP. The Framingham offspring study. Design and preliminary data. Prev Med. (1975) 4:518–25. 10.1016/0091-7435(75)90037-7 1208363

[24] Rimm EB Giovannucci EL Stampfer MJ Colditz GA Litin LB Willett WC. Reproducibility and validity of an expanded self-administered semiquantitative food frequency questionnaire among male health professionals. Am J Epidemiol. (1992) 135:1114–26. 10.1093/oxfordjournals.aje.a116211 1632423

[25] Kreger BE Splansky GL Schatzkin A. The cancer experience in the Framingham Heart Study cohort. Cancer. (1991) 67:1–6. 10.1002/1097-0142(19910101)67:1<1::aid-cncr2820670102>3.0.co;2-w 1845934

[26] Kannel WB Belanger A D'Agostino R Israel I. Physical activity and physical demand on the job and risk of cardiovascular disease and death: the Framingham study. Am Heart J. (1986) 112:820–5. 10.1016/0002-8703(86)90480-1 3766383

[27] Yiannakou I Pickering RT Yuan M Singer MR Moore LL. Potato consumption is not associated with cardiometabolic health outcomes in Framingham Offspring Study adults. J Nutr Sci. (2022) 11:e73. 10.1017/jns.2022.65 36117546 PMC9453580

[28] Cade JE Taylor EF Burley VJ Greenwood DC. Does the Mediterranean dietary pattern or the Healthy Diet Index influence the risk of breast cancer in a large British cohort of women? Eur J Clin Nutr. (2011) 65:920–8. 10.1038/ejcn.2011.69 21587285

[29] Couto E Sandin S Löf M Ursin G Adami H-O Weiderpass E. Mediterranean dietary pattern and risk of breast cancer. PLoS ONE. (2013) 8:e55374. 10.1371/journal.pone.0055374 23390532 PMC3563544

[30] Nkondjock A Ghadirian P. Diet quality and BRCA-associated breast cancer risk. Breast Cancer Res Treat. (2007) 103:361–9. 10.1007/s10549-006-9371-0 17063275

[31] Pot GK Stephen AM Dahm CC Key TJ Cairns BJ Burley VJ. Dietary patterns derived with multiple methods from food diaries and breast cancer risk in the UK Dietary Cohort Consortium. Eur J Clin Nutr. (2014) 68:1353–8. 10.1038/ejcn.2014.135 25052230 PMC4340564

[32] Dela Cruz R Park S-Y Shvetsov YB Boushey CJ Monroe KR Le Marchand L. Diet quality and breast cancer incidence in the Multiethnic Cohort. Eur J Clin Nutr. (2020) 74:1743–7. 10.1038/s41430-020-0627-2 32286532 PMC7554070

[33] Fung TT Rexrode KM Mantzoros CS Manson JE Willett WC Hu FB. Mediterranean diet and incidence of and mortality from coronary heart disease and stroke in women. Circulation. (2009) 119:1093–100. 10.1161/CIRCULATIONAHA.108.816736 19221219 PMC2724471

[34] Demetriou CA Hadjisavvas A Loizidou MA Loucaides G Neophytou I Sieri S. The Mediterranean dietary pattern and breast cancer risk in Greek-Cypriot women: a case-control study. BMC Cancer. (2012) 12:113. 10.1186/1471-2407-12-113 22443862 PMC3323439

[35] Mourouti N Kontogianni MD Papavagelis C Plytzanopoulou P Vassilakou T Malamos N. Adherence to the Mediterranean diet is associated with lower likelihood of breast cancer: a case-control study. Nutr Cancer. (2014) 66:810–7. 10.1080/01635581.2014.916319 24847911

[36] Zaragoza-Martí A Cabañero-Martínez M Hurtado-Sánchez J Laguna-Pérez A Ferrer-Cascales R. Evaluation of Mediterranean diet adherence scores: a systematic review. BMJ Open. (2018) 8:e019033. 10.1136/bmjopen-2017-019033 29478018 PMC5855302

[37] Park Y-M Zhang J Steck SE Fung TT Hazlett LJ Han K. Obesity Mediates the Association between Mediterranean diet consumption and insulin resistance and inflammation in US adults. J Nutr. (2017) 147:563–71. 10.3945/jn.116.243543 28298537 PMC5368583

[38] Feskanich D Rimm EB Giovannucci EL Colditz GA Stampfer MJ Litin LB. Reproducibility and validity of food intake measurements from a semiquantitative food frequency questionnaire. J Am Diet Assoc. (1993) 93:790–6. 10.1016/0002-8223(93)91754-E 8320406

