# Supplementary material for: Indices of Mediterranean diet adherence and breast cancer risk in a community-based cohort
Source: Front Nutr. 2023 Mar 21;10:1148075. doi: 10.3389/fnut.2023.1148075 (PMC10070722; doi:10.3389/fnut.2023.1148075)
Supplement: Supplementary file 1 [file Data_Sheet_1.docx]

Supplementary Material

Supplementary Table 1 Food items classified in each score component.

|  | **MDS index** | **aMED index** | **MeDiet index** | **MSDP index** |
| --- | --- | --- | --- | --- |
| **Vegetables except potatoes** | Tomatoes, tomatoes juice & sauce, broccoli, cabbage / coleslaw, cauliflower, Brussels sprouts, carrot, mixed vegetables, winter squash, eggplant / zucchini / summer squash, spinach, kale / mustard / chard, iceberg lettuce, romaine lettuce, celery, beets, alfalfa sprouts, garlic, and corn. | Tomatoes, tomatoes juice & sauce, broccoli, cabbage / coleslaw, cauliflower, Brussels sprouts, carrot, mixed vegetables, winter squash, eggplant / zucchini / summer squash, spinach, kale / mustard / chard, iceberg lettuce, romaine lettuce, celery, beets, alfalfa sprouts, garlic, and corn. | Tomatoes, tomatoes juice & sauce, broccoli, cabbage / coleslaw, cauliflower, Brussels sprouts, carrot, mixed vegetables, winter squash, eggplant / zucchini / summer squash, spinach, kale / mustard / chard, iceberg lettuce, romaine lettuce, celery, beets, alfalfa sprouts, garlic, corn, string beans, peas or lima beans, vegetables from pizza, other vegetables including radish, horseradish, pickles, green / red / stuffed pepper, vegetable soup, mushroom, onion, parsley, carrot juice, and vegetable juice. | Tomatoes, tomatoes juice & sauce, broccoli, cabbage / coleslaw, cauliflower, Brussels sprouts, carrot, mixed vegetables, winter squash, eggplant / zucchini / summer squash, spinach, kale / mustard / chard, iceberg lettuce, romaine lettuce, celery, beets, alfalfa sprouts, garlic, string beans, red chili sauce, vegetables from pizza, other vegetables including radish, horseradish, pickles, green / red / stuffed pepper, vegetable soup, mushroom, onion, parsley, carrot juice, and vegetable juice. |
| **Potatoes** | _ | _ | Baked / boiled / mashed potatoes, French fries, corn chips, yam, and sweet potatoes. | Baked / boiled / mashed potatoes, corn, yam, and sweet potatoes. |
| **Legumes** | Soybean / tofu, string beans, peas, and beans. | Soybean / tofu, string beans, peas, and beans. | Soybean / tofu, beans, and lentils. | _ |
| **Olives, pulses, and nuts** | _ | _ | _ | Peas / lima beans, beans, lentils, soybean / tofu, nuts, olives,and sunflower seeds. |
| **Fruits, fruit juice, and nuts** | Raisins / grapes, prunes, bananas, cantaloupe, watermelon, apples / pears, oranges, orange juice, grapefruit, grapefruit juice, strawberry, blueberries, peaches / apricot / plums, apple juice, other fruit juice, nuts, and peanut butter. | _ | _ | _ |
| **Fruits and fruit juice** | _ | Raisins / grapes, prunes, bananas, cantaloupe, watermelon, apples / pears, oranges, orange juice, grapefruit, grapefruit juice, strawberry, blueberries, peaches / apricot / plums, apple juice, and other fruit juice. | Raisins / grapes, prunes, bananas, cantaloupe, watermelon, apples / pears, oranges, orange juice, grapefruit, grapefruit juice, strawberry, blueberries, peaches / apricot / plums, apple juice, other fruit juice, other fruits including avocado, pineapple, kiwi fruit, raspberries, dates, figs, lemon juice, dried apricot, canned pineapple, and black olives. | Raisins / grapes, prunes, bananas, cantaloupe, watermelon, apples / pears, oranges, orange juice, grapefruit, grapefruit juice, strawberry, blueberries, peaches / apricot / plums, other fruits including avocado, pineapple, kiwi fruit, raspberries, dates, figs, lemon juice, dried apricot, canned pineapple, and black olives. |
| **Nuts** | _ | Peanut butter and nuts. | _ | _ |
| **Cereals** | Ready-to-eat cereals, cooked cereals, crackers, bread, rice, other grains, wheat germ, bran, popcorn, and pasta. |  | _ | _ |
| **Wholegrains** | _ | Wholegrain ready-to-eat cereals, cooked cereals, dark bread, brown rice, other grains, wheat germ, bran, and popcorn. | _ | Cooked oatmeal, breakfast cereals with ≥25% wholegrain or bran content by weight, dark bread, brown rice, a mixture of bulgur, kasha and couscous, popcorn, bran, wheat germ, granola bar, cooked oat bran, and graham crackers. |
| **Wholegrains, nuts, and seeds** | _ | _ | Cooked oatmeal, breakfast cereals with ≥25% wholegrain or bran content by weight, dark bread, brown rice, a mixture of bulgur, kasha and couscous, peanut butter, nuts, popcorn, sunflower seeds, and graham crackers. | _ |
| **Dairy (low and full fat)** | Whole milk, skim or low-fat milk, yogurt, cheese including cottage / ricotta cheese, American or cheddar, whipped / coffee cream, sour cream, and cheese cream. | _ | _ | Whole milk, skim or low-fat milk, regular & non-fat yogurt, cheese including cottage / ricotta cheese, American or cheddar, whipped / coffee cream, sour cream, cheese cream, and cheese from pizza. |
| **Full fat dairy** | _ | _ | Whole milk, regular yogurt, cheese including cottage / ricotta cheese, American or cheddar, and cheese from pizza. | _ |
| **Fish** | Canned tuna, dark fish including salmon, sardines, bluefish or swordfish, other fish, shrimp / shellfish / lobster. | Canned tuna, dark fish including salmon, sardines, bluefish or swordfish, other fish, shrimp / shellfish / lobster. | Canned tuna, dark fish including salmon, sardines, bluefish or swordfish, other fish, shrimp / shellfish / lobster. | Canned tuna, dark fish including salmon, sardines, bluefish or swordfish, other fish, shrimp / shellfish / lobster. |
| **Meat and meat products** | Hot dogs, deli meat, bacon, hamburger, meat sandwich or casserole, main meat dish, and chicken. | _ | _ | _ |
| **Red and processed meats** | _ | Hot dogs, deli meat, bacon, hamburger, meat sandwich or casserole, main meat dish. | _ | Hot dogs, deli meat, bacon, hamburger, meat sandwich or casserole, main meat dish, liver, beef soup. |
| **Red, processed meats and eggs** | _ | _ | Hot dogs, deli meat, bacon, hamburger, meat sandwich or casserole, meat main dish, liver, eggs, scrambled eggs, beef soup. | _ |
| **Poultry** | _ | _ | Chicken and chicken soup. | Chicken and chicken soup. |
| **Eggs** | _ | _ | _ | Egg and egg scramblers. |
| **Alcoholic beverages** | Wine, beer, “light” beer, and liquor. | Wine, beer, “light” beer, and liquor. | _ | Red and white wine. |
| **Sweets** | _ | _ | _ | Desserts such as cookies, brownies, doughnuts, cake, sweet-roll, pie, rice cake, figs cookies, sherbet / ice milk, ice cream, and pudding. Sweetened beverages, including cola (regular and decaffeinated), all other carbonated beverages with sugar, and non-carbonated fruit drinks, including lemonade and Hawaiian punch. Other sweets include candy bars, chocolate, candy without chocolate, jam / jelly / syrup / honey, and added sugar in beverages. |
| **Oils** | _ | _ | Types of oils were categorized into three categories: olive oil, mixed olive oil with other vegetable oils, and all other vegetable oils. | Types of oils were categorized into three categories: olive oil, mixed olive oil with other vegetable oils, and all other vegetable oils. |

Abbreviations: MDS, Mediterranean Diet Score index by Trichopoulou et al., (2003); aMED, Alternate Mediterranean Diet index by Fung et al., (2005); MeDiet, Mediterranean Diet index by Panagiotakos et al., (2006); and MSDP, Mediterranean-Style Dietary Pattern index by Rumawas et al., (2009).

**Supplementary Table 2** Pearson correlation coefficients in total scores for the four Mediterranean diet indexes.

|  | **MDS index** | **aMED index** | **MeDiet index** | **MSDP index** |
| --- | --- | --- | --- | --- |
| **MDS index** | 1 | 0.83 | 0.57 | 0.39 |
| **aMED index** |  | 1 | 0.61 | 0.54 |
| **MeDiet index** |  |  | 1 | 0.61 |
| **MSDP index** |  |  |  | 1 |
| Abbreviations: MDS, Mediterranean Diet Score index by Trichopoulou et al., (2003); aMED, Alternate Mediterranean Diet index by Fung et al., (2005); MeDiet, Mediterranean Diet index by Panagiotakos et al., (2006); and MSDP, Mediterranean-Style Dietary Pattern index by Rumawas et al., (2009). | | | | |
